# Evaluating the Impact of Individualized Interventions on Diabetes Distress and Glycemic Outcomes: A Shift From Glycated Hemoglobin (HbA1c) to Random Blood Sugar (RBS) in a Quasi-experimental Study

**DOI:** 10.7759/cureus.80890

**Published:** 2025-03-20

**Authors:** N L Swathi, Venu Priyanka P, Devadarshini M, Adnaan Parvez Mohammed, Jothieswari D, Muhammad Umar, Syed Muhammad Ali, Javed Iqbal, Pravallika P

**Affiliations:** 1 Department of Pharmacy Practice, Jawaharlal Nehru Technological University Anantapur, Chittoor, IND; 2 Department of Chemistry, Sri Venkateswara College of Pharmacy, Chittoor, IND; 3 Medicine, Khairpur Medical College, Khairpur, PAK; 4 Surgery, Weill-Cornell Medical School, Doha, QAT; 5 Acute Care Surgery, Hamad General Hospital, Doha, QAT; 6 Nursing, Hamad General Hospital, Doha, QAT; 7 Department of Pharmacy Practice, Sri Venkateswara College of Pharmacy, Chittoor, IND

**Keywords:** diabetes distress, glycemic control, psychological intervention, random blood sugar, type 2 diabetes mellitus

## Abstract

Background

Diabetes distress (DD) is a significant barrier to effective diabetes management, impacting self-care behaviors and glycemic control. While most studies utilize glycated hemoglobin (HbA1c) as a standard marker for glycemic regulation, cost constraints often limit its availability. This study explores the shift from HbA1c to random blood sugar (RBS) as an alternative measure and assesses the impact of individualized interventions on DD and glycemic outcomes in individuals with uncontrolled type 2 diabetes mellitus (T2DM).

Methods

A quasi-experimental study was conducted on 180 participants aged 18-65 years, divided into an experimental group (n=82) receiving structured psychological and lifestyle counseling and a control group (n=98) receiving standard care. DD and RBS levels were recorded at baseline and after three months of the intervention. Statistical analyses included Pearson’s correlation, student’s t-test, and Wilcoxon signed-rank tests to evaluate changes in distress levels and glycemic control.

Results

Post-intervention, the experimental group showed a significant reduction in DD (t = 15.26, p < 0.001, Cohen’s d = 1.685) and RBS (mean reduction = 10.68%), confirming the effectiveness of the structured interventions. The control group exhibited an unexpected increase in DD (t = -8.75, p < 0.001, Cohen’s d = -0.960), whereas RBS remained largely unchanged (1.29% increase). A significant correlation (p = 0.000) between DD reduction and RBS improvement was observed.

Conclusions

Individualized interventions significantly reduced diabetes distress and improved glycemic outcomes, demonstrating that RBS may serve as a cost-effective alternative to HbA1c. Future research should focus on directly comparing RBS and HbA1c levels and evaluating the long-term sustainability of the intervention benefits across different age groups.

## Introduction

Diabetes mellitus (DM) is a chronic metabolic disorder affecting millions of people worldwide, necessitating lifelong self-management through medication adherence, lifestyle modification, and psychological resilience [1]. However, a major barrier to optimal diabetes care is diabetes distress (DD), which is the emotional burden associated with disease management, fear of complications, and daily self-care demands [2]. Research indicates that 40-50% of individuals with type 2 diabetes mellitus (T2DM) experience DD, which significantly impacts treatment adherence, glycemic control, and the overall quality of life. If left unmanaged, DD contributes to higher HbA1c levels, an increased risk of complications, and poor long-term health outcomes [1,3]. Reduced distress can enhance self-efficacy, empowering individuals to actively engage in diabetes self-management. Psychological relief from distress fosters better problem-solving skills, allowing individuals to adhere more effectively to dietary recommendations, medication regimens, and lifestyle modifications. Moreover, lower distress levels are associated with improved emotional regulation, reduced avoidance behaviors, and enhanced motivation for consistent diabetes care [1-3].

While HbA1c remains the gold standard for assessing glycemic control, its high cost and limited accessibility pose challenges, particularly in resource-constrained settings such as India. Random blood sugar (RBS), a more accessible and cost-effective alternative, could serve as a substitute for assessing glycemic improvements linked to DD reduction. However, few studies have investigated the validity of the RBS in diabetes distress interventions, necessitating further research [4-6].

This study aimed to evaluate the effectiveness of individualized interventions in reducing diabetes distress and improving glycemic outcomes in patients with uncontrolled T2DM. The interventions included personalized counseling, dietary modifications, structured physical activity, and stress management techniques, such as yoga, all tailored to individual patient needs. Additionally, this study investigated the correlation between DD reduction and RBS levels, assessing whether RBS can serve as a reliable and cost-effective alternative to HbA1c. By identifying age- and sex-related variations in distress response, this study seeks to provide evidence-based strategies for personalized diabetes care and support the integration of holistic, patient-centered approaches in diabetes management.

Personalized interventions, including psychological support, structured counseling, and stress management techniques, such as yoga, play a crucial role in reducing diabetes distress (DD) and improving glycemic outcomes. Psychological and behavioral interventions, such as cognitive behavioral therapy and psychoeducational programs, have been shown to significantly reduce DD and enhance self-management behaviors by improving treatment adherence and coping mechanisms [1,2]. Additionally, yoga and stress management techniques have demonstrated benefits in reducing DD and improving metabolic parameters, including glycemic control, by enhancing stress resilience and promoting better physiological regulation [3-6]. Given the increasing recognition of psychosocial factors in diabetes management, incorporating these interventions can offer a holistic approach to improving both emotional well-being and glycemic outcomes.

## Materials and methods

This study evaluated the sustainability of glycemic improvements following personalized interventions, particularly assessing the shift from HbA1c to RBS as a cost-effective alternative. It also examines age-related variations in diabetes distress, intervention response, and treatment adherence, and explores the role of psychological support in medication compliance and patient satisfaction. Finally, this study aims to compare the effectiveness of RBS and HbA1c in distress management to determine their suitability for assessing glycemic outcomes across different age groups in the future.

**Figure 1:**
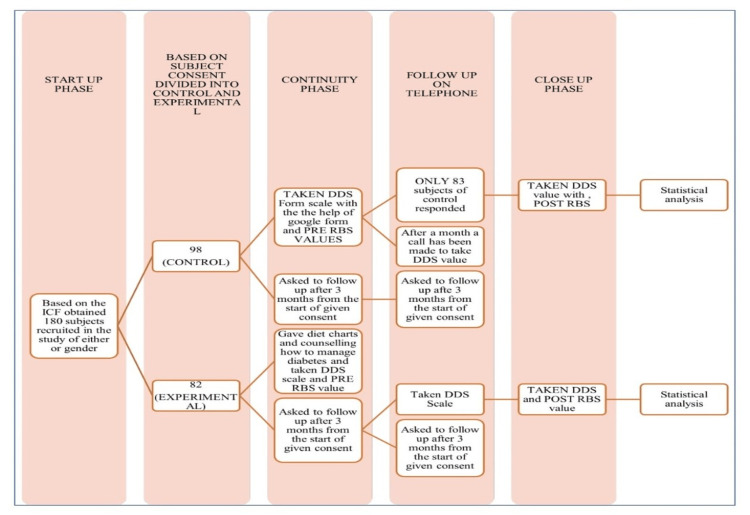
Flow chart describing the study methodology This flowchart outlines the study phases for evaluating diabetes distress (DDS) and random blood sugar (RBS) levels. A total of 180 participants were recruited and divided into control (n = 98) and experimental (n = 82) groups. Start-Up Phase: Baseline DDS and RBS values were collected. Continuity Phase: The control group received standard care while the experimental group received diet counseling and diabetes management guidance. Follow-Up Phase: DDS reassessments were conducted via telephone, with 83 control participants responding. Close-Up Phase: Post-intervention DDS and RBS values were recorded for statistical analysis.

The study recruited 180 participants aged 18-65 with confirmed uncontrolled diabetes (RBS > 200 mg/dL and a physician-confirmed diagnosis of uncontrolled type 2 diabetes mellitus (T2DM); Appendix A) who were willing to sign an informed consent form (Appendix B) and excluded those with gestational diabetes, severe comorbidities, insulin use, or different types of diabetes. The participants were categorized into two groups based on their level of willingness: the control group comprised 98 participants (54.44%), whereas the experimental group included 82 participants (45.55%). The study utilized RBS levels and the Diabetes Distress Scale (DDS) to assess four diabetes distress regimens using the Problem Areas in Diabetes Survey (PAID) scoring system [7]. The ratings of all individuals were documented. Participants who expressed willingness to make lifestyle changes, receive guidance on their food (Appendix C), maintain their mental health, and control their diabetes were assigned to the experimental group. In the experimental group, a clinical pharmacist gave participants 15-30 minutes of counseling (Appendix D). The control group was not subjected to any interventions (Figure 1).

In the follow-up phase, 15 individuals in the control group were excluded from the study because of declining participation due to personal reasons. This resulted in 83 subjects (50.33%) remaining in the control group while the experimental group still had 82 (49.696%) out of the original 165 subjects after dropout. After three months, the remaining participants were re-evaluated to measure any changes by recording their DDS and RBS values. One month after the initial follow-up, an experimental participant was contacted by phone and reminded to follow up three months after the initial consent.

Post-RBS measurements and final DDS values were obtained for both groups. Statistical analyses were performed to ascertain the effects of the interventions. The implementation of a systematic method that includes complete data collection and consistent follow-ups for a full examination of the impact of lifestyle adjustments on diabetes distress and blood sugar levels in comparison with the control group. The Institutional Ethics Committee of RVS Hospitals and Research Foundation issued ethical approval number IEC/RVSIMS/2023/05/06.

## Results

A gender distribution analysis in this study revealed a total of 68 (41.21%) females and 97 (58.78%) males, indicating a significant gender gap (Table 1). The type of involvement in this study was divided into the experimental and control groups, with 82 (49.69%) and 83 (50.30%) participants included in each group to ensure a balanced distribution for comparison analysis (Table 1).

**Table 1:** Distribution of gender and subjects

Category	Count (%)
*Gender Distribution*	
Female	68 (41.21%)
Male	97 (58.78%)
Subject Division	
Control	83 (50.30%)
Experiment	82 (49.69%)

Table 2 presents the Pearson correlation analysis between DDQ scores and RBS levels before (Pre) and after (Post) an intervention or observation period. The correlation values ranged from very weak (VW) to weak (W), indicating that the relationship between DDQ and RBS was generally low. A p-value of < 0.005 was considered statistically significant, and the significance status was marked as S (Significant) or NS (Not Significant) in the final column.

**Table 2:** The Pearson correlation values between the assessment scale and RBS VW - very weak, W - weak, S - significant, NS - not significant, P-values < 0.005 considered significant, RBS - random blood sugar, DDQ - diabetes distress questionnaire

S.NO	Pearson Correlation Value		Relation		Significance Value (P value)		Impression of Significance	
DDQ	Pre-RBS	Post-RBS	Pre-RBS	Post-RBS	Pre-RBS	Post-RBS	Pr- RBS	Post-RBS
1	0.185	0.263	VW	W	0.018	0.001	S	S
2	0.057	0.208	VW	W	0.467	0.007	NS	S
3	0.215	0.159	W	VW	0.005	0.042	S	S
4	0.056	0.171	M	VW	0.474	0.028	NS	S
5	0.154	0.196	VW	VW	0.048	0.012	S	S
6	0.147	0.247	VW	W	0.059	0.001	NS	S
7	0.144	0.233	VW	W	0.065	0.003	NS	S
8	0.047	0.209	VW	W	0.546	0.007	NS	S
9	0.063	0.165	VW	VW	0.422	0.034	NS	S
10	0.249	0.220	W	W	0.001	0.005	S	S
11	0.052	0.199	VW	VW	0.510	0.010	NS	S
12	0.169	0.159	VW	VW	0.030	0.041	S	S
13	0.111	0.203	VW	W	0.155	0.009	NS	S
14	0.123	0.152	VW	VW	0.114	0.052	NS	NS
15	0.031	0.125	VW	VW	0.697	0.110	NS	NS
16	0.165	0.098	VW	VW	0.035	0.210	S	NS
17	0.049	0.115	VW	VW	0.533	0.140	NS	NS
18	0.106	0.088	VW	VW	0.175	0.260	NS	NS
19	0.153	0.148	VW	VW	0.050	0.058	S	NS
20	0.052	0.234	VW	W	0.503	0.002	NS	S
21	0.196	0.251	VW	W	0.012	0.001	S	S
22	0.193	0.187	VW	VW	0.013	0.016	S	S
23	0.190	0.195	VW	VW	0.015	0.012	S	S
24	0.172	0.058	VW	VW	0.027	0.456	S	NS
25	0.006	0.266	VW	W	0.935	0.001	NS	S
26	0.096	0.150	VW	VW	0.222	0.054	NS	NS

Most correlation values were low, with Pearson coefficients ranging between 0.006 and 0.266, suggesting that DDQ scores have only a minimal association with RBS. Despite the weak correlations, several relationships were statistically significant, such as in rows 10 (Pre: 0.249, Post: 0.220, p < 0.005) and 21 (Pre: 0.196, Post: 0.251, p < 0.005). This indicates that even a weak correlation can be statistically meaningful when the sample size and variability are considered. In some cases, the correlation strength increased post-intervention (e.g., Row 1: Pre 0.185 → Post 0.263), while in others, it decreased (e.g., Row 4: Pre 0.056 → Post 0.171). However, a number of correlations remained non-significant, particularly those with high p-values (e.g., Row 14: Pre 0.123, Post 0.152, p > 0.005).

Table 3 presents the diabetic distress scores for both the control and experimental groups, measured before (Pre) and after (Post) the intervention stratified according to age (in Years). Before the intervention, the control group exhibited low to moderate distress levels, with younger participants (Age Codes 1-2, corresponding to ages 18-30 years) generally reporting lower distress scores while middle-aged participants (Age Codes 3-4, ages 31-50 years) exhibited moderate distress levels. Older participants (Age Code 5-6, ages 51-65 years) had varied distress levels, with some showing moderate distress and others reporting lower levels. After the intervention, some control participants, especially those from the younger and middle-aged groups (in years), showed reductions in distress, but older participants exhibited more stable distress levels, indicating minimal overall improvement.

**Table 3:** Diabetes distress scores for pre and post-intervention in control and experimental groups -- age stratified DDS: diabetes distress scores

Age in Years	Control Scores		Experimental Scores	
Age Code	Pre-Control DDS	Post-Control DDS	Pre-Experimental DDS	Post-Experimental DDS
1	33	0	70	0
2	32	43	43	24
3	16	31	52	25
4	26	33	53	29
5	28	35	51	24
6	24	36	54	30
7	25	33	53	27
8	23	33	56	25
9	27	28	53	26
10	29	35	50	28

In contrast, the experimental group consistently exhibited higher pre-intervention distress scores across all age groups, with younger participants (Age Codes 1-2) showing moderate to high distress and middle-aged (in years) and older participants (Age Codes 3-6) reporting significantly high distress levels. However, post-intervention scores declined across all age groups (in years), demonstrating that the intervention was effective in reducing distress levels, particularly among middle-aged and older participants, who had the highest baseline distress. The age (in years) coding system allowed for a structured analysis, enabling researchers to observe age-related variations in distress scores and the intervention’s effectiveness across different age brackets (age in years), with greater reductions observed in older and middle-aged individuals than in younger participants.

Table 4 examines the relationship between RBS reports and DDS averages, assessing statistical significance using p-values (< 0.005 considered significant). Before the intervention, the p-value (0.009) was not significant, indicating no meaningful association between distress level and blood sugar. However, post-intervention, the p-value (0.000) was significant, suggesting a strong relationship between reduced distress and changes in the blood sugar levels of the participants. This finding highlights the potential impact of diabetes distress on blood sugar regulation and warrants further investigation in the future.

**Table 4:** Pre and post-random blood sugar (RBS) reports and diabetes distress scores (DDS): significance

Category	Pre-Intervention RBS (Mean)	Post-Intervention RBS (Mean)	Percentage Change (%)	p-value	Significance
Control Group	274.41	277.94	+1.29%	0.009	Not Significant
Experimental Group	291.66	260.50	-10.68%	0.000	Significant

Table 5 presents the results of paired statistical tests comparing pre- and post-intervention diabetic distress scores for both the experimental (E) and control (C) groups. The analysis included the student's t-test and the Wilcoxon signed-rank test, both of which assessed whether there was a statistically significant difference between the pre and post-scores.

**Table 5:** Analysis of pre- and post-intervention diabetic distress scores CI = confidence interval, SE = standard error, df = degrees of freedom

Category	Test Statistic	df	p-value	Mean Difference	SE Difference	95% CI (Lower)	95% CI (Upper)	Effect Size
Experimental Group (E)	Student’s t	15.26	81.0	26.5	1.73	23.0	29.9	Cohen’s d = 1.685
	Wilcoxon W	3351	NA	26.5	1.73	23.0	30.0	Rank biserial correlation = 0.969
Control Group (C)	Student’s t	-8.75	82.0	-16.6	1.90	-20.4	-12.8	Cohen’s d = -0.960
	Wilcoxon W	257	NA	-16.5	1.90	-20.0	-12.5	Rank biserial correlation = -0.849

For the experimental group (E), the t-test (t = 15.26, p < 0.001) showed a highly significant increase in distress reduction, with a mean difference of 26.5 and a large effect size (Cohen’s d = 1.685). The Wilcoxon test (W = 3351, p < 0.001) confirmed this result, with a rank biserial correlation of 0.969, indicating a strong effect of the intervention.

In contrast, the control group (C) exhibited an unexpected trend, with distress scores increasing post-intervention. The t-test (t = -8.75, p < 0.001) and Wilcoxon test (W = 257, p < 0.001) showed a significant negative mean difference (-16.6) with a moderate negative effect size (Cohen’s d = -0.960). The rank biserial correlation (-0.849) further confirmed this shift.

These findings suggest that while the intervention was effective in reducing distress in the experimental group, the control group’s distress unexpectedly increased, indicating the need to investigate potential external influences on distress levels in the absence of intervention. The observed increase in DDS among the control group may be attributed to several factors. First, the lack of structured psychological support likely contributed to sustained or worsening distress levels, as participants did not receive targeted interventions to address diabetes-related emotional burdens. Second, limitations of usual care, where routine diabetes management does not necessarily include distress-reduction strategies, may have reinforced feelings of frustration and a perceived lack of progress. Lastly, a nocebo effect could have played a role, where awareness of study participation without receiving an intervention led to heightened distress in some individuals. Similar findings have been reported in behavioral diabetes research, where a lack of active engagement in psychological interventions exacerbated distress levels in control groups. Future studies should consider additional support mechanisms for control participants to mitigate this effect.

## Discussion

This study evaluated the impact of individualized interventions, including counseling, dietary modifications, structured physical activity, and stress management (yoga), on DD and glycemic control in individuals with T2DM. Unlike previous studies that primarily assessed glycemic outcomes using HbA1c, our study explored the feasibility of using RBS as a cost-effective alternative in resource-limited settings. The findings demonstrated that post-intervention DD scores significantly decreased in the experimental group (t = 15.26, p < 0.001, Cohen’s d = 1.685), along with a notable reduction in RBS levels (mean reduction = 10.68%). Conversely, the control group exhibited an unexpected increase in the DD (t = -8.75, p < 0.001, Cohen’s d = -0.960), whereas the RBS levels remained largely unchanged (1.29% increase). A significant correlation (p = 0.000) between DD reduction and improved glycemic control was observed, reinforcing the growing evidence that psychosocial interventions play a crucial role in diabetes self-management.

Impact of psychological interventions on DD and glycemic control

Our findings align with those of multiple studies demonstrating the effectiveness of psychological and behavioral interventions in reducing DD and improving treatment adherence. Abbas et al. (2023) conducted a randomized controlled trial (RCT) to assess the impact of cognitive behavioral therapy (CBT) on diabetes distress and glycemic control, reporting a significant DD reduction (F = 222.710, p < 0.001) and improved treatment adherence (F = 67.579, p < 0.001) [8]. Similarly, the TELE-DD trial (Lozano del Hoyo et al., 2022) found that a telephonic psychoeducational intervention reduced HbA1c from 8.72% to 7.03% (p < 0.001) and improved DD and medication adherence over 18 months [9]. Our study supports these findings by demonstrating a significant decline in DD following personalized interventions, reinforcing the role of psychosocial support in diabetes care.

Diabetes distress, treatment adherence, and glycemic control

Several studies have emphasized the negative correlation between DD and treatment adherence. Hoogendoorn et al. (2021) (GRADE study) found that a one-unit increase in DD resulted in a 2.07-point decrease in medication adherence (p < 0.0001), with significant HbA1c worsening over time [10]. Additionally, Roddy et al. (2023) (FAMS 2.0 RCT) demonstrated that reducing distress not only improved self-care behaviors but also enhanced the well-being of patients and their support persons [11]. These results parallel our findings, where a significant reduction in the DD was associated with improved glycemic outcomes, indicating that effective distress management can enhance treatment adherence and self-care in diabetes. To achieve this, we assessed changes in DDS and glycemic control post-intervention, comparing our findings with existing literature on diabetes distress management. Additionally, we explored age-related variations in DDS reduction and evaluated the feasibility of RBS as a practical alternative to HbA1c for glycemic monitoring.

Age and therapy-related variations in DD reduction

Our study categorized participants into different age brackets using an age coding system, revealing that middle-aged and older individuals (ages 31-65 years) experienced greater DD reductions than younger participants. This aligns with the findings of Liu et al. (2020), who analyzed diabetes distress across different therapy types and found that insulin-treated individuals had higher distress (p < 0.05), while those on combination therapy had lower distress and greater happiness levels (p < 0.05) [5]. The EMPOWER study (Cummings et al., 2017) further supported this by demonstrating that women with reduced DD had a mean HbA1c reduction of 0.34%, while those with increased distress had a 0.2% increase in HbA1c (p = 0.05) [12]. Our findings suggest that individualized distress-reducing interventions are particularly beneficial for older adults and those with higher baseline distress levels.

Use of RBS vs. HbA1c as a glycemic measure

Most studies assessing distress-related glycemic changes have relied on HbA1c as the primary biomarker, whereas our study explored RBS as an alternative due to cost constraints. Previous trials, including those by Crowley et al. (2022) [13] (Telehealth RCT) and Guo et al. (2022) [14] (Mindfulness RCT), demonstrated significant HbA1c reductions of 1.59% and 0.8%, respectively, following the intervention. The INDEPENDENT trial (Rutten et al., 2020) further validated the effectiveness of collaborative care models in improving depressive symptoms and reducing HbA1c levels [15]. While our study found a significant correlation between DD reduction and RBS improvement, further direct comparisons between RBS and HbA1c are needed to confirm their reliability as glycemic markers.

Veteran and population-specific considerations

Population-based differences in distress management have also been explored in previous studies. Lewinski et al. (2024) investigated diabetes distress in U.S. veterans with T2DM and found that regimen distress, emotional burden, and provider-related factors were major contributors [16]. Similarly, Cummings et al. (2017) (EMPOWER study) identified sociodemographic disparities in distress reduction among African American women with uncontrolled diabetes [12]. Although our study did not focus on a specific demographic subgroup, the findings highlight the need for culturally tailored interventions to maximize distress reduction across diverse populations.

## Conclusions

This study demonstrated that individualized interventions, including counseling, dietary modifications, structured physical activity, and stress management, significantly reduced diabetes distress (DDS) and improved random blood sugar (RBS) levels in individuals with type 2 diabetes. The findings support the role of psychosocial interventions in diabetes care and suggest that RBS could serve as a cost-effective alternative to HbA1c for glycemic monitoring. Despite these promising results, the study has certain limitations, including the absence of HbA1c measurements, which limits direct comparisons with RBS, and a short follow-up duration (three months), restricting long-term assessment. Additionally, self-reported DDS scores may introduce response bias, and the study's sample size was geographically limited, reducing generalizability. Future research should directly compare RBS and HbA1c in diabetes distress interventions, extend follow-up periods, and explore age and gender-specific responses. Incorporating digital health interventions, mobile-based counseling, and remote monitoring tools may further enhance patient adherence and long-term engagement in distress management.

## Figures and Tables

**Figure 2:**
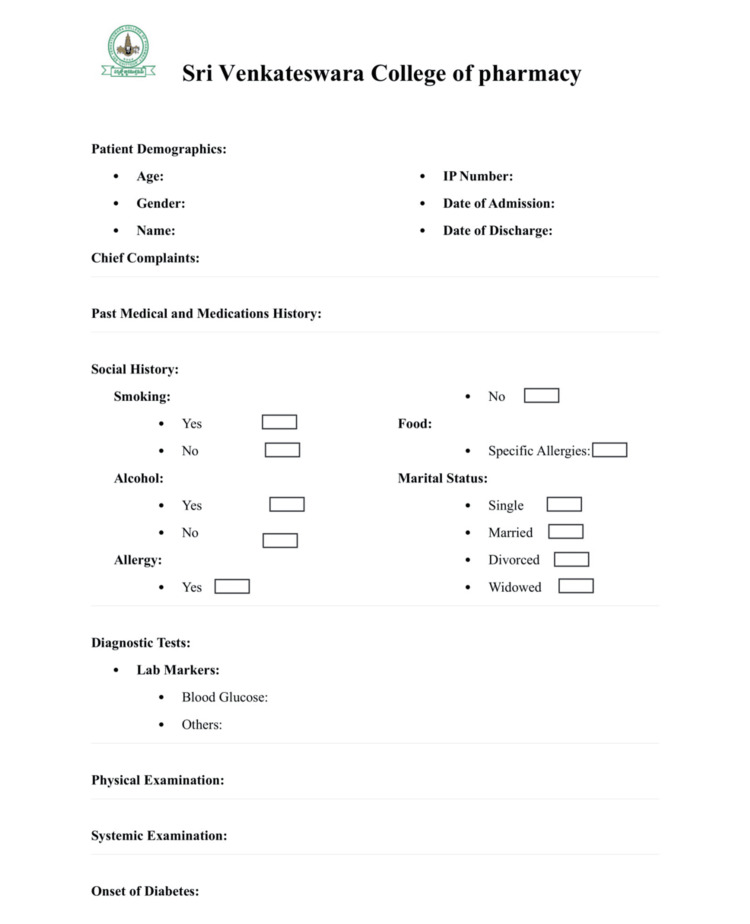
Subject information documentation form

**Figure 3:**
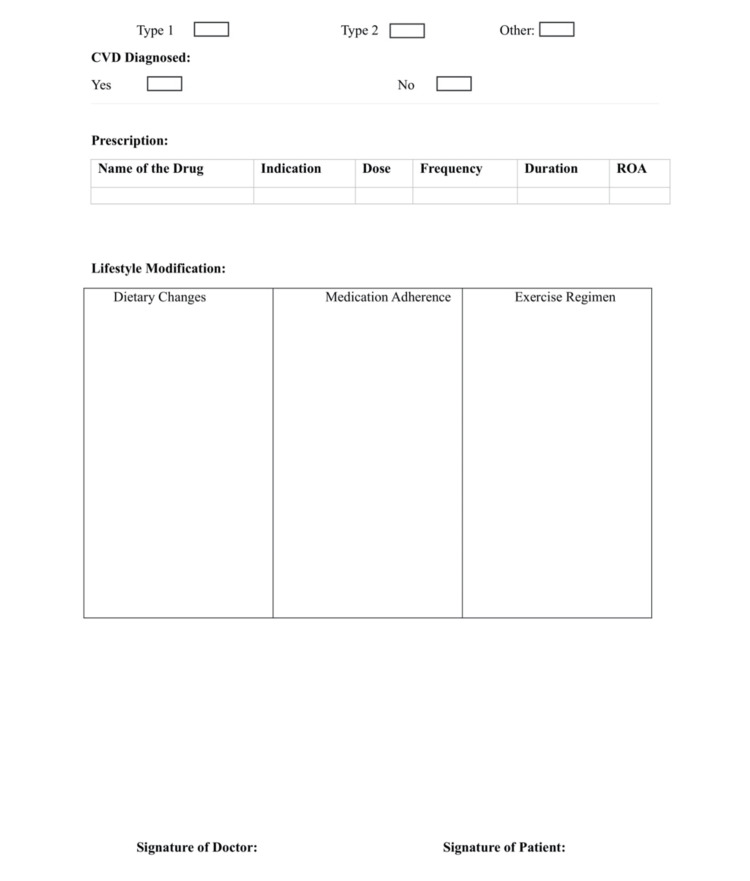
Subject information documentation form (Contd.)

**Figure 4:**
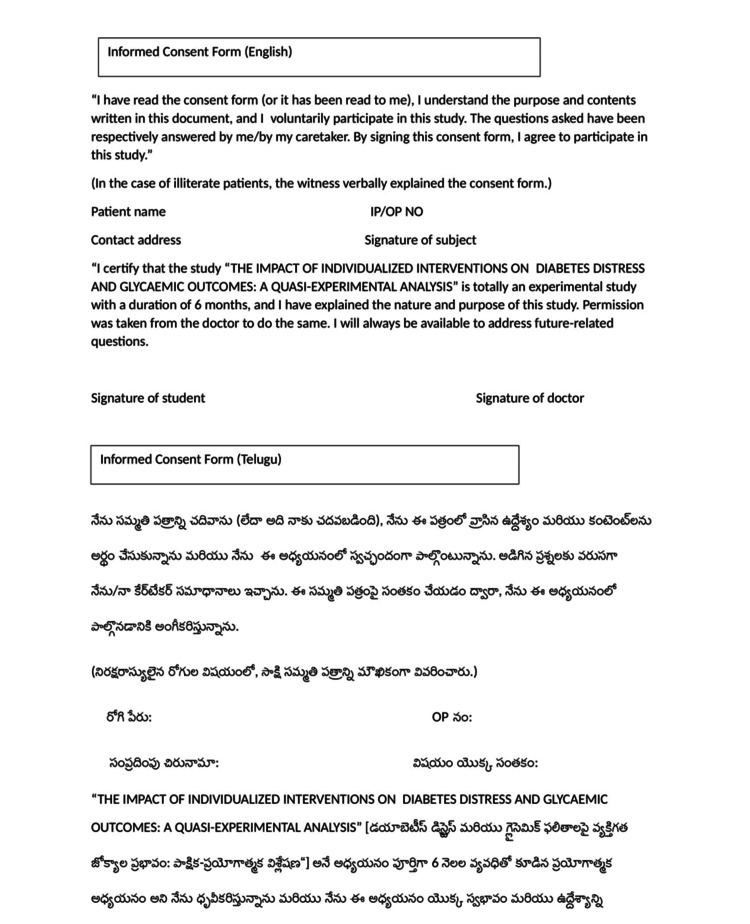
Informed consent form in the native language (Telugu) and English

**Figure 5:**
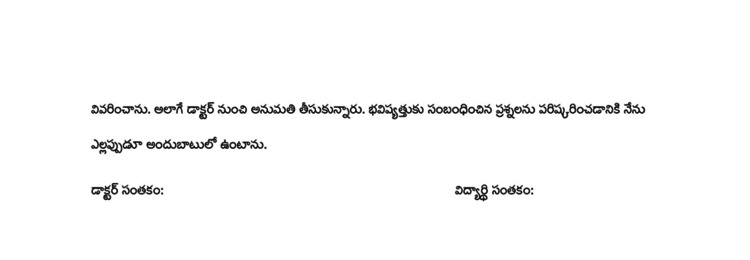
Informed consent form in the native language (Telugu) and English

**Figure 6:**
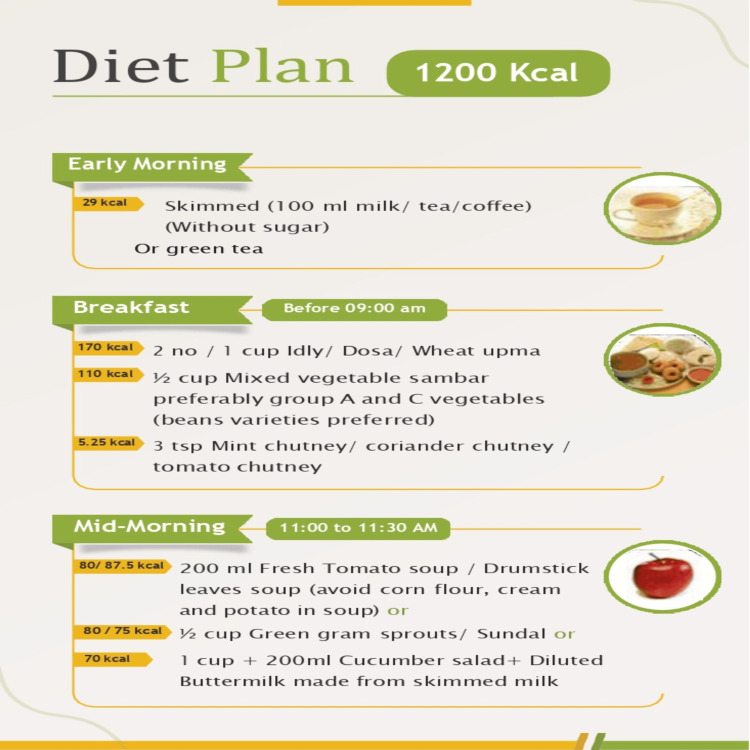
Diet plan

**Figure 7:**
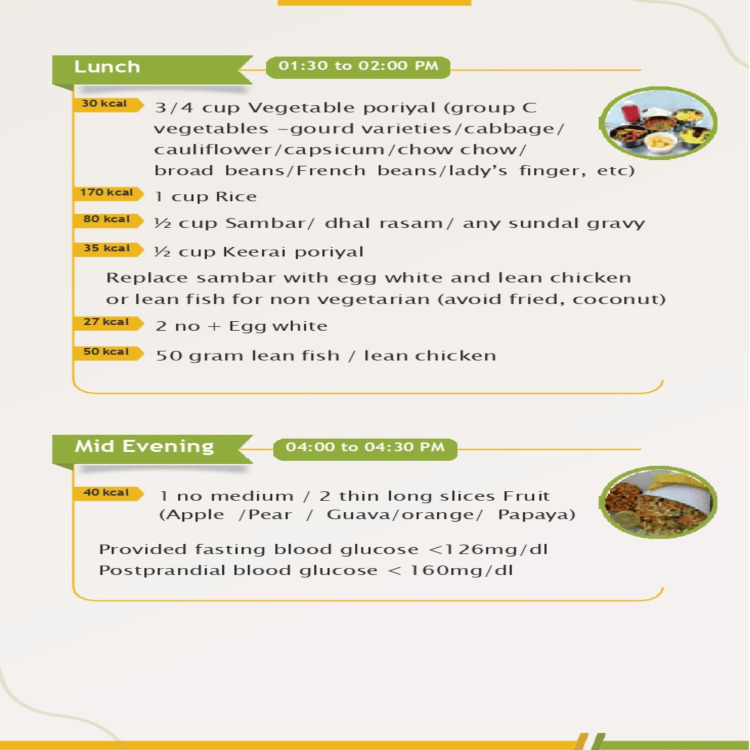
Diet plan (Contd. 1)

**Figure 8:**
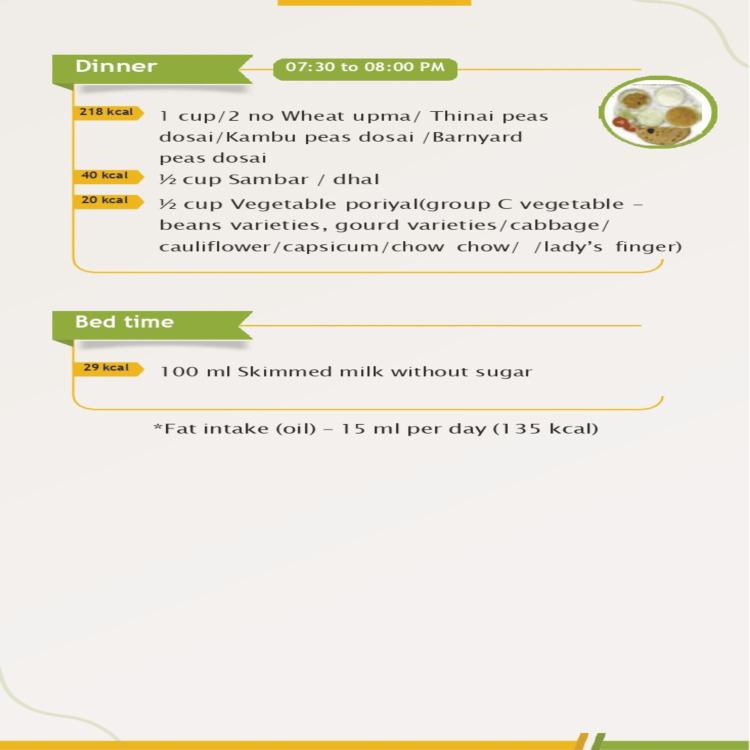
Diet plan (Contd. 2)

**Figure 9:**
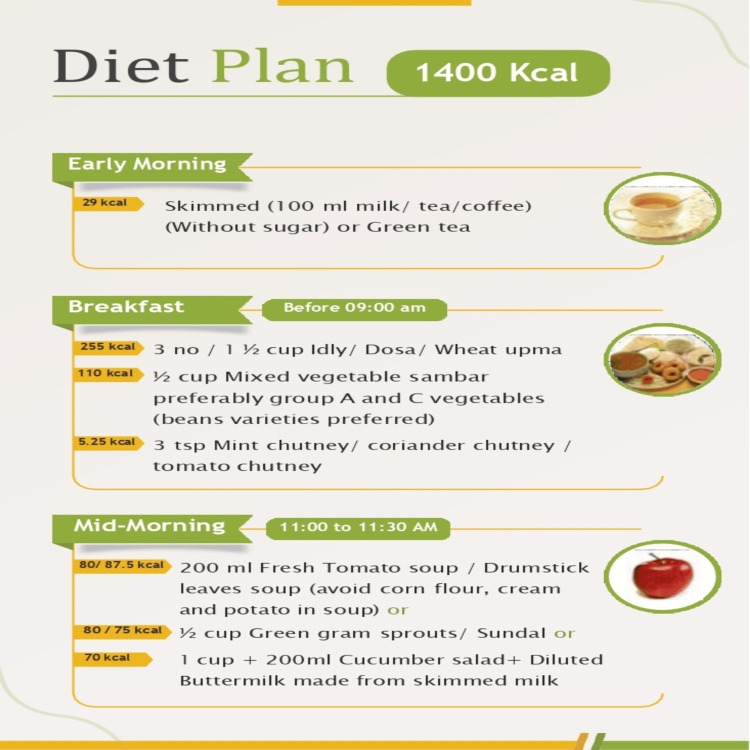
Diet plan (Contd. 3)

**Figure 10:**
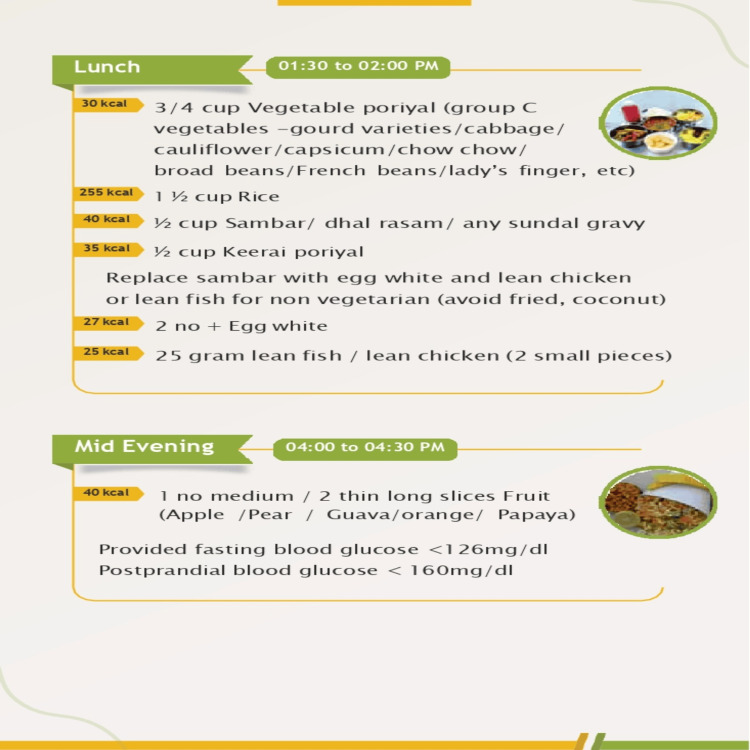
Diet plan (Contd. 4)

**Figure 11:**
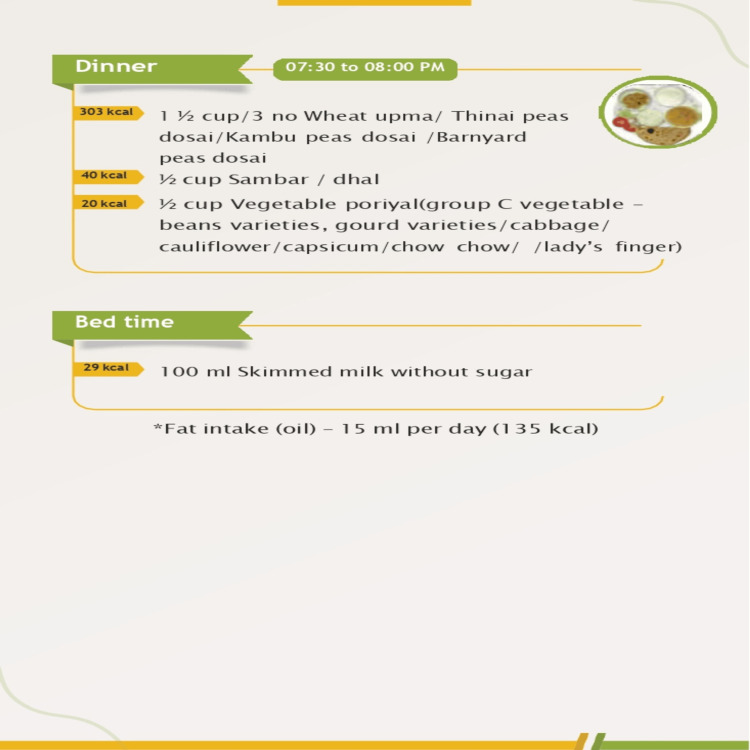
Diet plan (Contd. 5)

**Figure 12:**
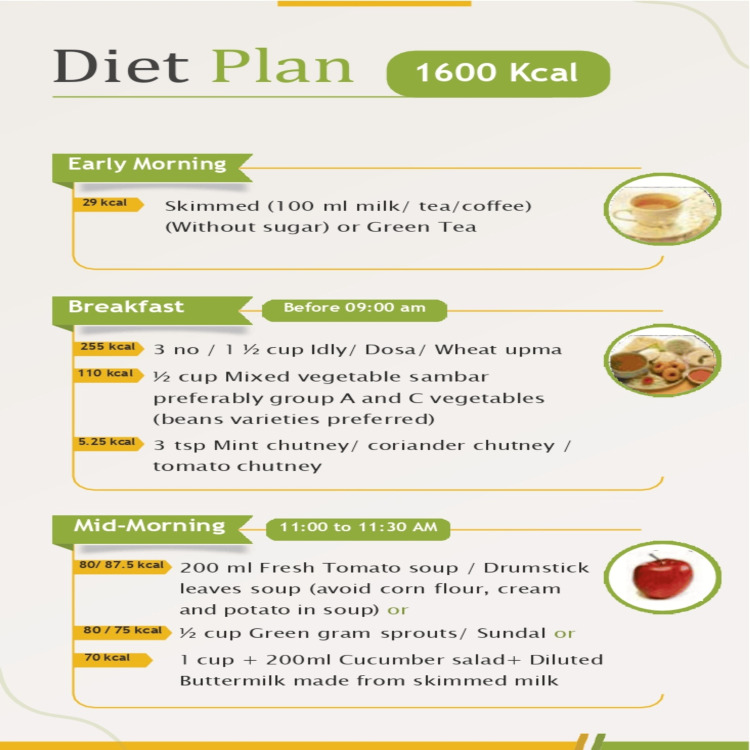
Diet plan (Contd. 6)

**Figure 13:**
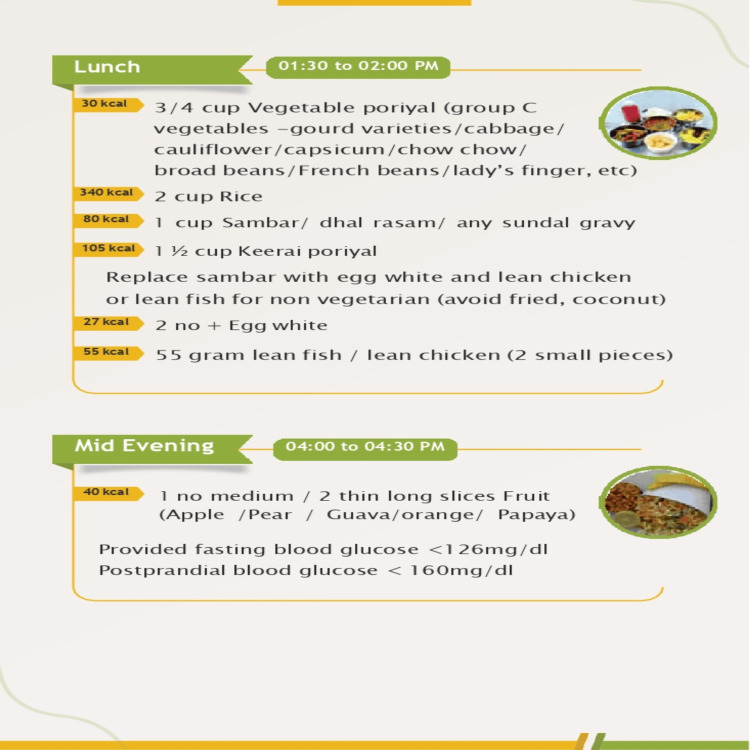
Diet plan (Contd. 7)

**Figure 14:**
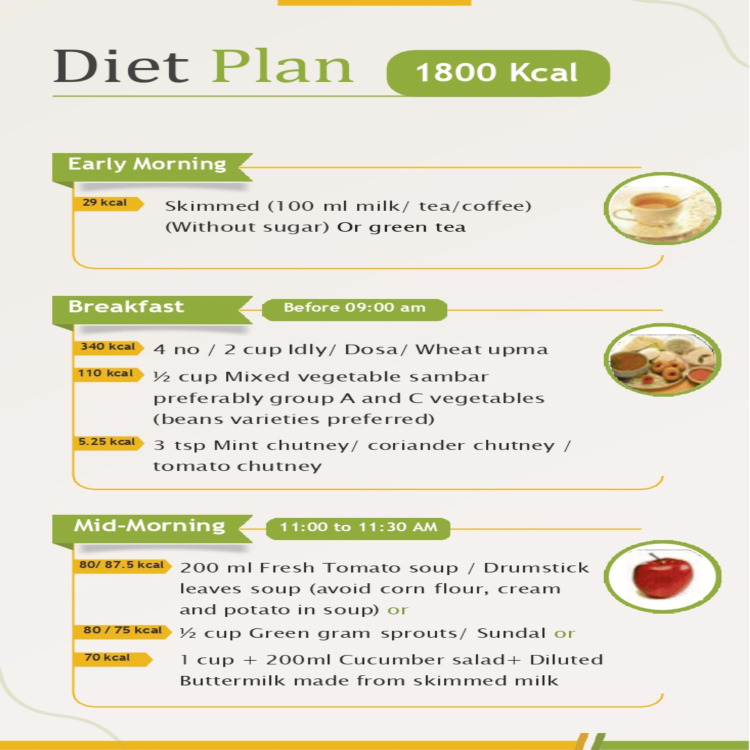
Diet plan (Contd. 8)

**Figure 15:**
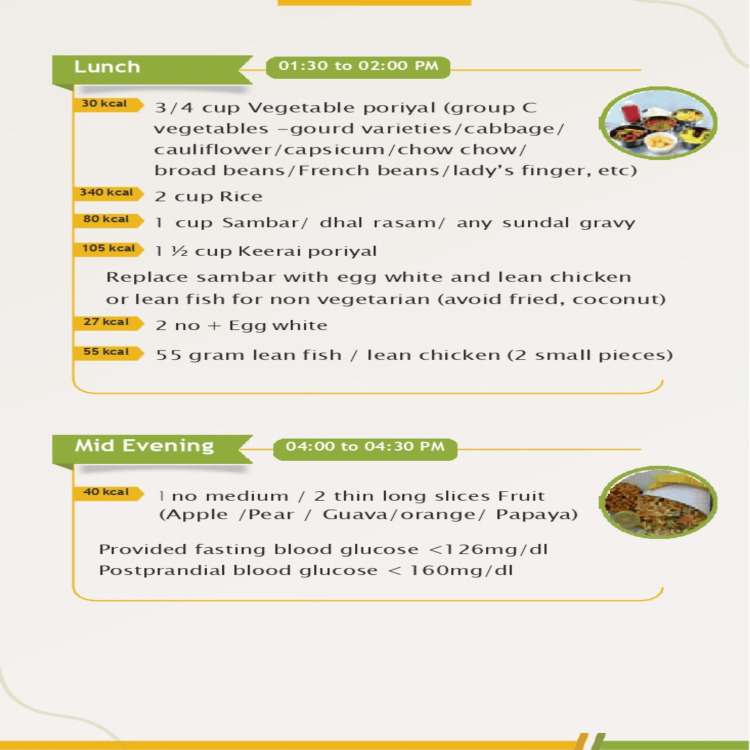
Diet plan (Contd. 9)

**Figure 16:**
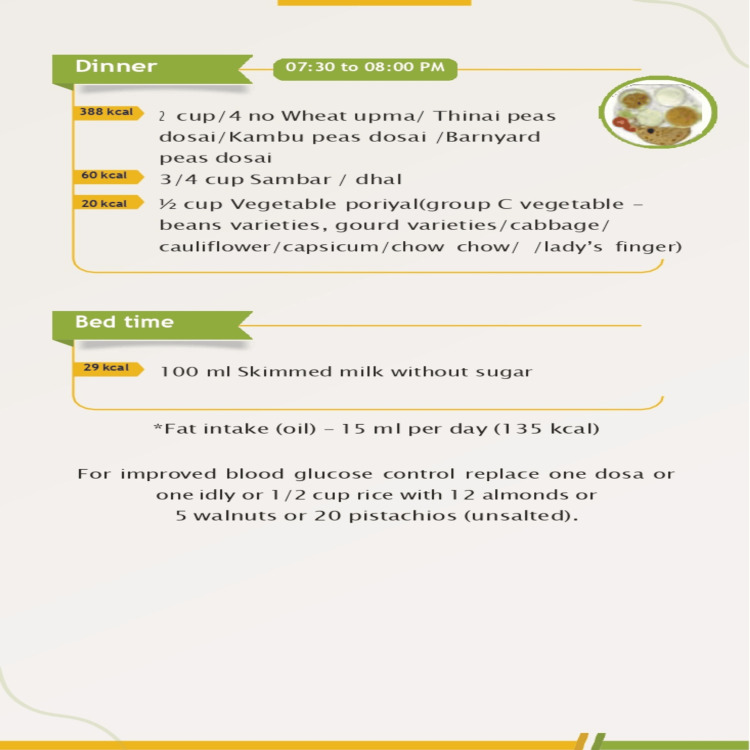
Diet plan (Contd. 10)

**Figure 17:**
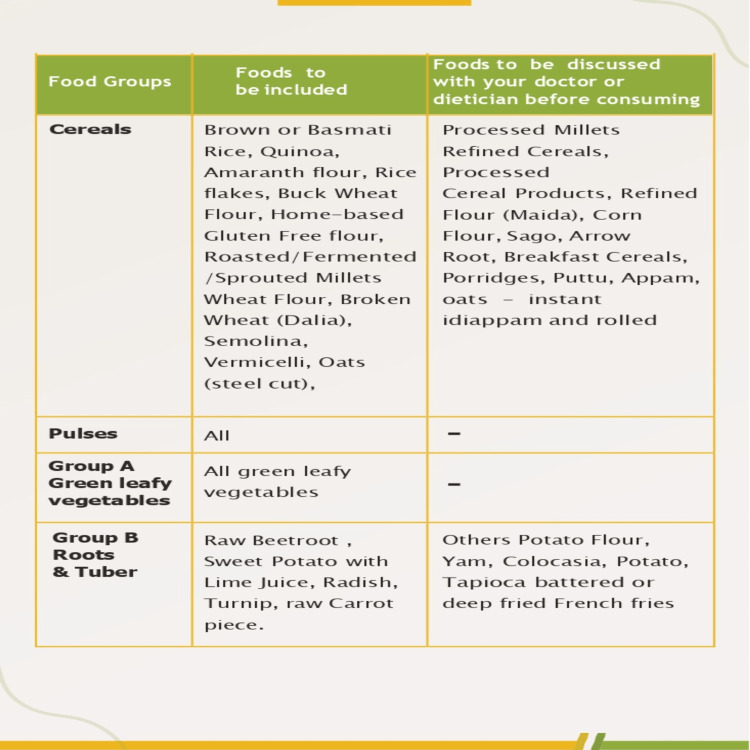
Diet plan (Contd. 11)

**Figure 18:**
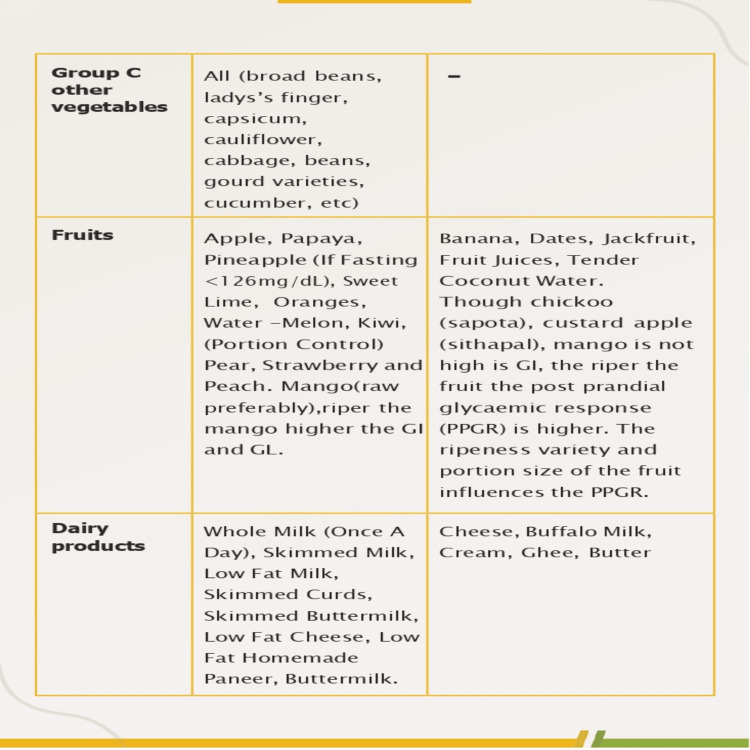
Diet plan (Contd. 12)

**Figure 19:**
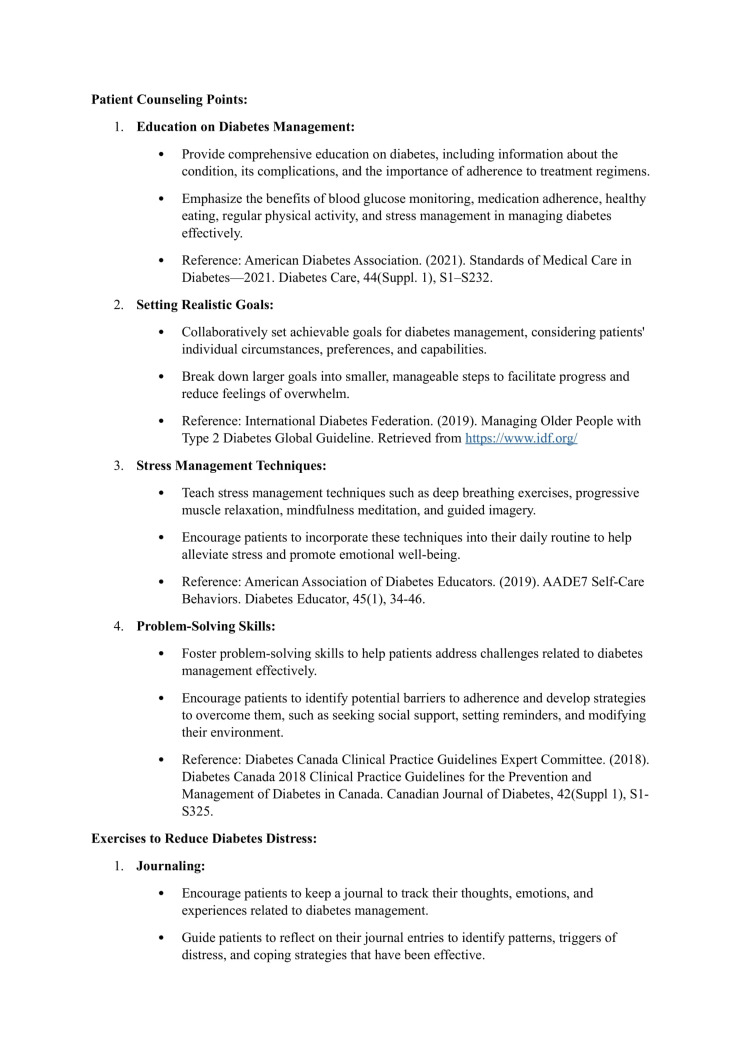
Subject counselling suggestions referred to conduct this study

**Figure 20:**
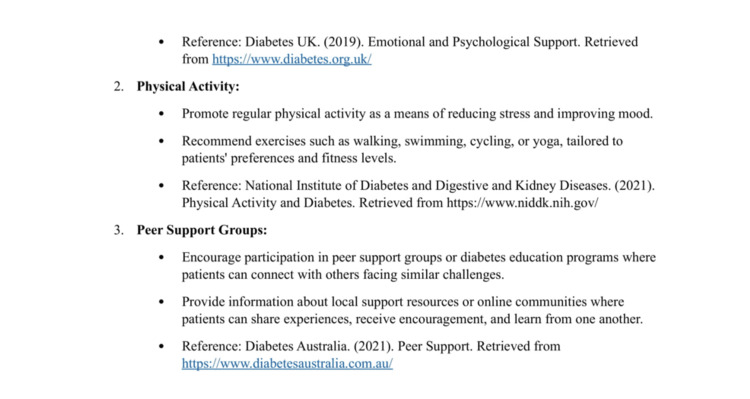
Subject counselling suggestions referred to conduct this study (Contd.)

## Appendices

Appendix A 

Appendix B 

Appendix C

Appendix D 
